# An Appeal

**Published:** 1884-09

**Authors:** 


### AN APPEAL.

There must be hundreds of copies of the INDEPENDENT PRACTITIONER for August, 1883, and January, 1884, that their owners have no special desire to preserve. Our files of both numbers are entirely exhausted, and we are meeting with extreme difficulty in obtaining enough to supply new subscribers who desire to commence their subscriptions with the year. To any one who will mail to us either of the missing numbers, we will send a copy of Dr. Miller's work on "Fermentation in the Human Mouth," with other valuable reprints, or we will pay liberally in cash, if preferred. Will not our friends try and help us out?

